# Extreme convergence in egg-laying strategy across insect orders

**DOI:** 10.1038/srep07825

**Published:** 2015-01-16

**Authors:** Julia Goldberg, Joachim Bresseel, Jerome Constant, Bruno Kneubühler, Fanny Leubner, Peter Michalik, Sven Bradler

**Affiliations:** 1 Johann-Friedrich-Blumenbach-Institute of Zoology and Anthropology, Georg-August-University Göttingen, Berliner Str. 28, 37073 Göttingen, Germany; 2 Royal Belgian Institute of Natural Sciences, Vautier Street 29, 1000 Brussels, Belgium; 3 Schädrütihalde 47c, 6006 Lucerne, Switzerland; 4 Zoological Institute and Museum, Ernst-Moritz-Arndt-University, Johann-Sebastian-Bach-Str. 11/12, 17489 Greifswald, Germany

## Abstract

The eggs of stick and leaf insects (Phasmatodea) bear strong resemblance to plant seeds and are commonly dispersed by females dropping them to the litter. Here we report a novel egg-deposition mode for Phasmatodea performed by an undescribed Vietnamese species of the enigmatic subfamily Korinninae that produces a complex egg case (ootheca), containing numerous eggs in a highly ordered arrangement. This novel egg-deposition mode is most reminiscent of egg cases produced by members of unrelated insect orders, e.g. by praying mantises (Mantodea) and tortoise beetles (Coleoptera: Cassidinae). Ootheca production constitutes a striking convergence and major transition in reproductive strategy among stick insects, viz. a shift from dispersal of individual eggs to elaborate egg concentration. Adaptive advantages of ootheca formation on arboreal substrate are likely related to protection against parasitoids and desiccation and to allocation of specific host plants. Our phylogenetic analysis of nuclear (28S, H3) and mitochondrial (COI, COII) genes recovered Korinninae as a subordinate taxon among the species-rich Necrosciinae with *Asceles* as sister taxon, thus suggesting that placement of single eggs on leaves by host plant specialists might be the evolutionary precursor of ootheca formation within stick insects.

Predation is a primary driving force in the evolution of insects, triggering elaborate anti-predator adaptations that involve diverse camouflage and reproductive strategies^1^,^2^,^3^. Among terrestrial arthropods, the herbivorous stick and leaf insects or Phasmatodea exhibit an exceptionally high degree of plant mimicry, imitating various parts of plants such as leaves, twigs and bark^4^. Camouflage, or more precisely masquerade^3^, already played a crucial role in the early evolution of this insect group^5^,^6^ and does not stop short at the insects' eggs, which have a strong resemblance to plant seeds^7^,^8^. The phasmatodean egg capsule is remarkably hard-shelled and diversely sculptured (Fig. 1a), bearing a lid-like operculum at its anterior pole through which the offspring emerges (Fig. 1b). Adult females lay eggs over a period of several months at a rate of one (or less) to several per day^4^,^8^. During oviposition females of most species remain in the foliage and drop or flick single eggs from their ovipositor to the ground^8^,^9^. Some species place their eggs more carefully by inserting them into crevices or soil, glue them to substrate or pierce them into leaves^8^,^9^,^10^,^11^. One common feature of these diverse egg-laying modes is that eggs are laid singly, with very few exceptions where separate eggs are arranged in loose clutches or in a small row^9^. Here we report the first stick insect to produce a complex egg case or ootheca that contains numerous eggs in a highly ordered arrangement. This unknown mode of egg deposition constitutes an unexpected evolutionary novelty and a major shift in the reproductive strategy of phasmatodeans, i.e. a switch from dispersal of individual eggs to sophisticated egg concentration.

## Results

### Egg case morphology

**Figure 1:**
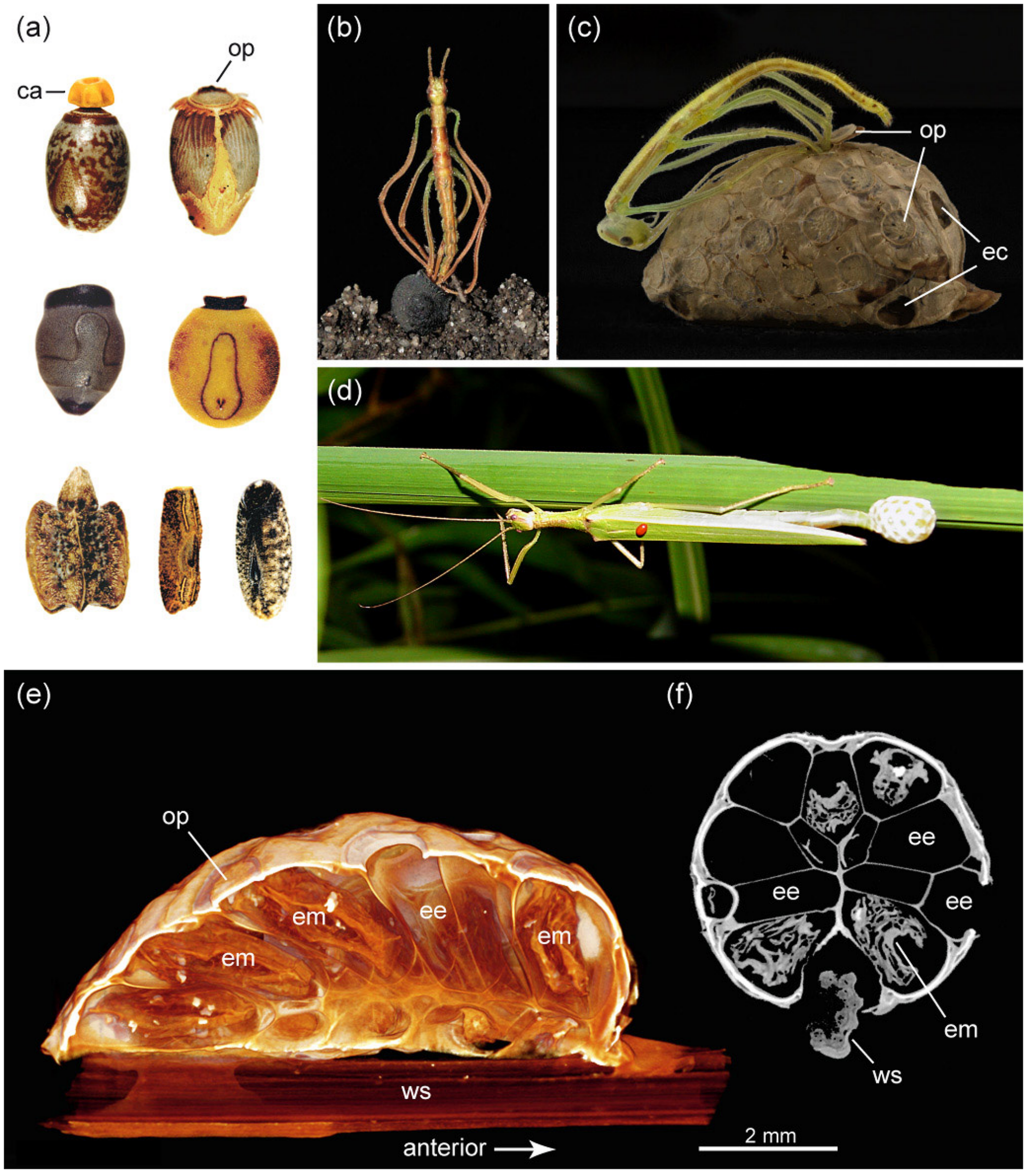
(a) single eggs of various stick insects (not to scale); (b) juvenile (*Anchiale* spec.) hatching from egg; (c) juvenile Korinninae spec. hatching from ootheca, please note that the opened operculum has a 2^nd^ outer layer consisting of ootheca material; (d) female Korinninae spec. while producing an ootheca; (e,f) ootheca scans: (e) longitudinal section of volume rendered ootheca showing arrangements of eggs, abandoned or with embryos inside; (f) cross section. ca, capitulum; ec, egg-like chamber; ee, empty egg; em, embryo; op, operculum; ws, wooden stick. Photographs taken by the authors.

The oothecae (Fig. 1c–f) have an oval general appearance. The scanned ootheca consists of 34 eggs oriented radially around the substrate center (twig or leaf) to which it was attached. Each egg's anterior end is directed to the ootheca's surface bearing the operculum. The posterior end of each egg is tapered and directed towards the ootheca's center, forming a honeycomb-like lattice with eggs appearing hexagonal or pentagonal in cross section. The egg capsule appears to be extraordinarily thin and must have been soft-walled during oviposition. There is some material of unknown origin filling the minor space between the tightly packed eggs and providing a fine but dense layer on the ootheca's surface, particularly around the opercular rim. There are four small chambers at the anterior and two at the posterior pole of the ootheca, which have an operculum-like opening but do not harbour eggs. The function of these egg-like chambers is enigmatic; ventilation is unlikely as their openings appear sealed by a thin layer of ootheca material (for further details see electronic supplementary movie).

### Identification of the ootheca-producing stick insect species

Based on the following combination of characters, we identified this ootheca-producing species as an undescribed member of the subfamily Korinninae, the most species-poor subfamily recognised among stick insects^12^,^13^,^14^,^15^,^16^: Legs with area apicalis, a demarcated triangular area located ventrally on the apex of the tibiae, and with non-pectinate ungues; hind wings fully developed with unbranched radius vein; female operculum with deeply notched hind margin. The subfamily was erected by Günther^12^ based on the genera *Korinnis* and *Kalokorinnis*, which currently comprise only seven described species from Borneo, Thailand and the Philippines^13^,^14^,^15^,^16^.

### Phylogenetic analysis

**Figure 2:**
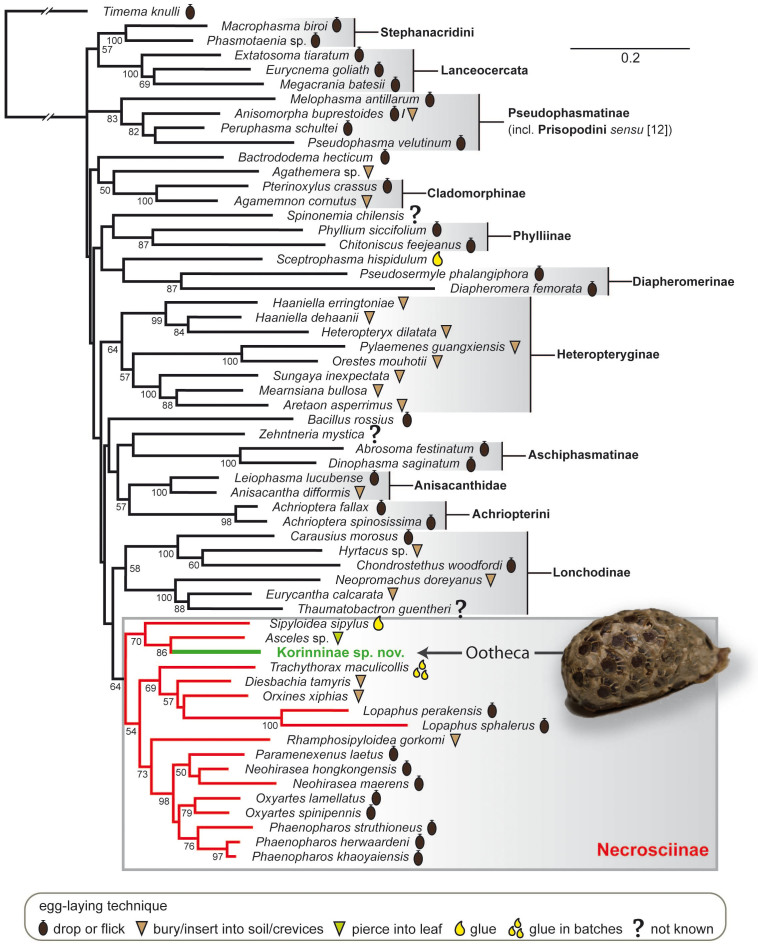
Maximum likelihood tree of the Phasmatodea based on combined molecular data with egg-deposition modes mapped on taxa according to symbol legend. Bootstrap values >50 are given below nodes. Ootheca photographed by Bruno Kneubühler.

**Figure 3:**
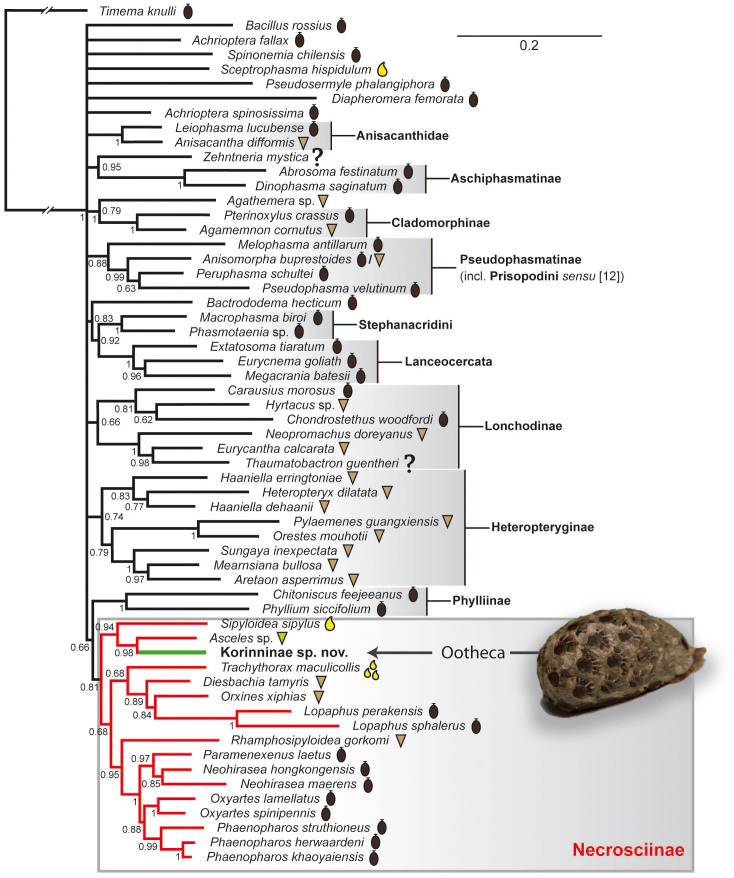
Majority-rule consensus tree of post-burn trees of Phasmatodea resulting from Bayesian analysis of the combined molecular data. Posterior probabilities are given below nodes. Symbols of egg-deposition modes as in Fig. 2. Ootheca photographed by Bruno Kneubühler.

Our analyses of the concatenated molecular data using likelihood (Fig. 2) and Bayesian (Fig. 3) methods yielded similar phylogenies consistent with previous studies^11^,^17^,^18^ including well-supported monophyletic Aschiphasmatinae, Cladomorphinae, Diapheromerinae, Heteropteryginae, Lanceocercata, Lonchodinae, Pseudophasmatinae (including *Melophasma*) and Stephanacridini. Furthermore, we found good support for Anisacanthidae and Achriopterini. The undescribed Korinninae species was recovered as a subordinate taxon within Necrosciinae with *Asceles* as sister taxon (MLB = 86; BPP = 0.98).

## Discussion

Concentrated egg-deposition in form of an ootheca is unique and highly unusual for stick insects. In contrast, ootheca formation is a defining groundplan feature of the Dictyoptera (cockroaches, termites and praying mantises)^19^,^20^,^21^, but is also found in various other insect groups such as grasshoppers and locusts (Orthoptera: Caelifera)^19^,^22^,^23^, heelwalkers (Mantophasmatodea)^24^,^25^ and even some chrysomelid beetles (Coleoptera: Cassidinae)^19^,^26^. Oothecae, also referred to as egg pods or egg cases, likely evolved to protect the eggs from desiccation, predators and parasitoids^19^,^20^,^23^,^25^. In arid environment, the eggs of grasshoppers are laid into the ground protected by a hardening foamy substance that is also adhesive to surrounding habitat material like sand and soil^23^. A similar mode of egg deposition is found in Mantophasmatodea who produce egg pods only when soil is provided^25^. The reported stick-insect ootheca is most reminiscent of those found in praying mantises (Mantodea) or tortoise beetles (Cassidinae) whose eggs do not bear a operculum, but who also build the egg case externally upon certain substrate, e.g. against plant parts or rocky underground, including external application of coating during and after highly ordered egg placement^19^,^20^,^26^.

Stick insects in general are well adapted to disperse their hard-shelled, seed-like eggs by dropping them individually to the ground, which is considered to represent the ground pattern in Phasmatodea^27^ and to be an advantageous strategy for cryptic animals^2^. On the contrary, in webspinners (Embioptera), which are the sister group of stick and leaf insects^18^,^28^,^29^,^30^, eggs are often deposited in tight or loose clusters within the silk galleries the individuals inhabit, usually attached to a substrate^31^,^32^. This behaviour also involves brood care and embedment in a hardened paste consisting of pulverised plant substrate and fecal pellets supplemented by salivary secretions^31^,^32^. In the Californian stick insect *Timema*, which represents one of the two basal lineages among extant Phasmatodea^18^,^27^, females also coat their eggs in pulverised substrate before dropping or placing single eggs onto the ground^33^.

In all remaining stick insects, the species-rich Euphasmatodea, females that lay single, non-adhesive eggs represent the plesiomorphic condition^27^ (see Fig. 2). Numerous phasmid species throw the eggs some distance in order to further disperse them and avoid clumping in the litter, thus decreasing susceptibility to predators and egg-parasitoids^4^. Additional adaptations promoting dispersal involve specialized structures attached to the egg's operculum, the capitulum (Fig. 1a), which induces egg removal and transportation by ants^7^,^8^. Density-responsive egg-parasitoids and predation by granivorous birds are considered to be significant driving forces for acquisition of these elaborate dispersal strategies^34^,^35^. Two subgroups of the chrysidids or cuckoo wasps, Amiseginae and Loboscelidiinae, are obligatory parasitoids to stick insect eggs^36^. The predominantly flightless female wasps search for eggs in low vegetation and leaf litter. They chew holes into the egg capsule with their specialised mouthparts and oviposit into the phasmatodean egg. The operculum of the egg, which is not damaged during this process, is burst open by the juvenile wasp after development is finished^36^. The geographic distribution of these wasps overlaps worldwide with those of Phasmatodea^36^, and parasitisation rates of eggs between 40 and 80% have been reported^8^. Egg deposition on arboreal substrate as performed by Korinninae and few other stick insect taxa might reduce parasitisation rates significantly^36^. Furthermore, the dense layer coating the surface of the ootheca likely provides further protection as does the tight egg arrangement that largely decreases the capsule surface which can be accessed by the parasitoids, since egg opercula remain unaffected. The reduced capsule surface might also reduce desiccation of eggs although the oothecae are not found in a particularly arid environment.

A further adaptive advantage of arboreal concentration of eggs is probably related to the insects' diet. Flightless phasmatodeans are exceedingly polyphagous, linked to their limited motility in diversely structured forests^37^. In contrast, volant forms as found in the species-rich Southeast Asian Necrosciinae who can more easily reach dispersed plants have a more restricted diet and are often regarded as host plant specialists^38^.

These potential advantages obviously compensate for the parental investment of a time-consuming ootheca production and for the drawback of synchronous egg hatch that places newly-hatched offspring in greater risk of being detected by predators.

We recovered the Korinninae species as a subordinate taxon within the Necrosciinae. This is particularly noteworthy, since Korinninae and Necrosciinae are considered to belong to the two different suborders of Phasmatodea, Areolatae and Anareolatae^12^. This traditional subdivision is based on the presence (areolate) or absence (anareolate) of the area apicalis on the tibiae, but neither Areolatae nor Anareolatae appear to be monophyletic^11^,^17^,^18^. Furthermore, the phylogenetic placement of the areolate Korinninae within the anareolate Necrosciinae suggests an atavistic origin or reversal of this trait in Korinninae, i.e. a recovery of the area apicalis after former loss, a phenomenon described before for wings and other morphological traits^18^,^39^. Originally considered to be an “isolated” areolate taxon without obvious relationships to other phasmatodean subfamilies^12^, recent classifications placed Korinninae either as sister group to the areolate Southeast Asian Aschiphasmatinae^13^ or as sister to the likewise areolate Neotropical Prisopodinae^14^. The results of our molecular analysis refute any close relationship of Korinninae to either of these groups, namely to *Abrosoma* + *Dinophasma* (Aschiphasmatinae) or to *Melophasma* (Prisopodinae).

A strong overall resemblance between Korinninae and certain Necrosciinae, both gracile winged stick insects with long antennae, was indicated before^12^. Flighted necrosciines can effectively distribute their offspring and often place eggs onto or near host plant leaves, sometimes even in small rows or batches^4^,^9^,^38^. The ootheca-forming Korinninae female exhibits good flight capability (pers. obs. J.B., J.C.) and appears to be a diet specialist as well since the offspring did not accept any plant offered in captivity (pers. obs. B.K.). Yet, the natural host plant range of Korinninae remains to be identified. The plant specialist *Asceles*, which was recovered as sister taxon to the Korinninae, pierces single eggs onto leaves^10^. Consequently, we consider the careful placement of eggs on host plants to be the evolutionary precursor of ootheca formation. However, the few oothecae collected were not found associated with a certain potential host plant.

The biology of Korinninae is virtually unknown and the egg deposition mode hitherto unreported. However, single eggs of *Kalokorinnis* were described before based on immature eggs removed from the dissected abdomen of a female^13^,^15^. The eggs were described and illustrated as being tapered towards their polar end, which might be an adaptation to the arrangement in egg cases. Thus, formation of an ootheca might apply to all Korinninae and constitute an apomorphic trait of this group.

Moreover, the subordinate phylogenetic placement of Korinninae within Necrosciinae provides insight into the evolutionary speed of this convergent transition of ootheca formation in stick insects, which requires numerous physiological and behavioural adaptations, such as synchronized egg maturation, development of specific accessory glands and ootheca-building capability. Females of some Necrosciinae genera such as *Calvisia*, *Marmessoidea* and *Trachythorax* glue their eggs in loose single-layer clusters without any coating on parts of plants^9^. With synchronous egg maturation and sticky glandular secretion already developed in these taxa, it would be straightforward to assume that these forms preceded the ootheca formation. However, we found no support for this assumption as our molecular phylogeny recovered *Trachythorax* to be unrelated to Korinninae (Figs. 2,3). Consequently, egg clumping on arboreal substrate, which is also reported for at least one member of Pseudophasmatinae (*Metriophasma* *diocles*)^9^, evolved several times independently within Phasmatodea. This mode of egg deposition is probably advantageous in the foliage in absence of flightless wasps that search for dispersed phasmid eggs on the ground. However, at least some winged Amiseginae were also found on trees^9^.

Admittedly, we do not know whether novel glands have been developed in Korinninae. The internal anatomy of the new species is not known, and a broad comparative investigation of female internal genitalia of phasmatodeans is lacking. In dictyopterans, the produced egg mass is coated with secretions from genitalic accessory glands of the ninth abdominal segment^40^. These glands might be part of the ground pattern of pterygote insects^41^, though absent in some taxa, i.e. Mantophasmatodea^24^, which appear closely related to Phasmatodea^30^ and also produce egg pods^25^. Paired glands leading into the bursa copulatrix (genital chamber) were described for some stick insects^42^, but were not found in *Eurycantha*^42^ and not reported for *Timema*^43^. Noteworthy, these accessory glands are also absent in *Calvisia* and *Trachythorax*, two taxa that glue their eggs in batches. Both species possess various other glands associated with the oviduct instead^42^, thus indicating that a derived egg deposition mode might indeed require anatomical modifications.

Necrosciinae has started a rapid radiation approximately 30 million years ago^11^, most probably giving rise to Korinninae and ootheca formation within less than 10 million years. Further phylogenetic investigations of phasmatodeans based on more densely sampled Necrosciinae diversity will become necessary to adequately describe the evolutionary transitional steps leading to ootheca formation.

In summary, parasitisation and host-plant specificity have most probably triggered the rapid evolution of this unique oviposition strategy among stick insects. Yet, stick insects remain poorly studied in their natural environment and our explanations await further investigations, particularly in regard of the ecology and reproductive anatomy of this enigmatic new species.

## Methods

### Collection of material

Three oothecae, four male and three female stick insects were collected during a field trip to Cat Tien National Park and Dong Nai Biosphere Reserve South Vietnam, July 2013. The oothecae of the undescribed species were found glued to different plant species and also on the wall of a guest house. Specimens are housed in the Royal Belgian Institute of Natural Sciences, Brussels, Belgium and in the Institute of Ecology and Biological Resources, Hanoi, Vietnam.

### Micro-computed tomography

The dry ootheca was mounted on a wooden stick and scanned with a Xradia MicroXCT-200 X-ray imaging system (Carl Zeiss X-ray Microscopy Inc., Pleasanton, USA) at 30 KV and 6 W (0.39 scintillator-objective lens unit, 3 s exposure time, 13.5 µm pixel size). The obtained data were processed using the 3D analysis software AMIRA v. 5.4.3 (Visage Imaging, Berlin, Germany).

### Phylogenetic analyses

The molecular analyses targeted mitochondrial (COI, COII) and nuclear gene regions (H3, 28S) that were used in previous Phasmatodea studies^11^,^17^,^18^. PCR cycling, purification, sequencing conditions and sequence alignments followed^11^,^44^. Phylogenetic analyses included 59 phasmatodean taxa (supplementary table S1) with the Californian *Timema* used as outgroup^11^,^17^ and were performed using the program Geneious (Geneious v7.0.1. Available at http://www.geneious.com). Alignments of different genetic markers were concatenated and subsequent analyses were performed using the combined dataset. We utilized likelihood (ML) and Bayesian algorithms (BI) for analyses. We employed the Akaike information criteria (AIC) as implemented in Modeltest v3.7^45^ to select a suitable model of sequence evolution for the combined data.

ML analysis used PHyML^46^ incorporating a GTR model with gamma-distributed rate variation across sites and a proportion of invariable sites. Bootstrap re-sampling used 500 iterations and resulting ML bootstrap values (MLB) were recorded.

MrBayes 3.1.2^47^ was utilized to implement Bayesian analysis, applying the GTR model with gamma-distributed rate variation across sites and a proportion of invariable sites. Analyses with MrBayes used four independent Markov Chain Monte Carlo (MCMC) runs for ten million generations with a burn-in of 25% and a tree sampling frequency of 1000. Resulting posterior probabilities on the nodes were recorded. Results were then checked for convergence. Trees sampled after burn-in of the four different MCMC runs were merged and used to construct a 50% majority rule consensus tree. Resulting posterior probabilities (BPP) were recorded. Novel sequence data generated in the present study are deposited on GenBank under the accession numbers KP300885-KP300930. Sequence data from previous studies can be accessed under accession numbers FJ474100–FJ474403^11^, KJ024376–KJ024575^17^, AY121129–AY121186 and AY125216–AY125326^18^.

## Supplementary Material

Supplementary Information Stick insect ootheca

Supplementary Information Supplementary Dataset 1
