# Supplementary material for: Extreme convergence in egg-laying strategy across insect orders
Source: Sci Rep. 2015 Jan 16;5:7825. doi: 10.1038/srep07825 (PMC4648384; doi:10.1038/srep07825)
Supplement: Supplementary Information — Supplementary Dataset 1 [file srep07825-s2.doc]

**Extreme convergence in egg-laying strategy across insect orders**

Julia Goldberg^1^, Joachim Bresseel^2^, Jerome Constant^2^, Bruno Kneubühler^3^, Fanny Leubner^1^, Peter Michalik^4^ & Sven Bradler^1^

^1^Johann-Friedrich-Blumenbach-Institute of Zoology and Anthropology, Georg-August-University Göttingen, Berliner Str. 28, 37073 Göttingen, Germany

^2^Royal Belgian Institute of Natural Sciences, Vautier Street 29, 1000 Brussels, Belgium

^3^Schädrütihalde 47c, 6006 Lucerne, Switzerland

^4^Zoological Institute and Museum, Ernst-Moritz-Arndt-University, Johann-Sebastian-Bach-Str. 11/12, 17489 Greifswald, Germany

Supplementary table 1. List of employed taxa. *Timema* is used as outgroup for euphasmatodean phylogeny. Euphasmatodea arranged in alphabetical order across “Areolatae” and “Anaraolatae”. Classification largely follows^11,12,17^. Redundant tribes (those with content identical to the corresponding subfamily) are omitted.

| Taxon | Subfamily / tribe | Family | ^*^Source of sequence data | Geographical locality |
| --- | --- | --- | --- | --- |
| Timematodea | | | | |
| *Timema knulli* Strohecker, 1951 | Timematinae | Timematidae | [18] | California |
| Euphasmatodea: ‘Areolatae’ | | | | |
| *Abrosoma festinatum* Brock & Seow-Choen, 1995 | Aschiphasmatinae: Aschiphasmatini | Aschiphasmatidae | [11] | Malaysia (culture) |
| *Agathemera* sp. | Agathemerinae | Agathemeridae | [11] | Chile |
| *Anisacantha difformis* (Redtenbacher 1906) | Anisacanthinae | Anisacanthidae | present study | Madagascar (culture) |
| *Anisomorpha buprestoides* (Stoll, 1813) | Pseudophasmatinae: Anisomorphini | Pseudophasmatidae | [11] | Babersville, Volusia County, Florida, USA |
| *Aretaon asperrimus* (Redtenbacher, 1906) | Heteropteryginae: Obrimini | Bacillidae | present study | Borneo (culture) |
| *Bacillus* *rossius* (Rossi, 1790) | Bacillinae: Bacillini | Bacillidae | [18] | France (culture) |
| *Chitoniscus feejeeanus* (Westwood, 1864) | Phylliinae | Phylliidae | [11] | Fiji |
| *Dinophasma saginatum* (Redtenbacher, 1906) | Aschiphasmatinae: Aschiphasmatini | Aschiphasmatidae | [11] | Borneo (culture) |
| *Haaniella* *dehaanii* (Westwood, 1859) | Heteropteryginae: Heteropterygini | Bacillidae | present study | Borneo (culture) |
| *Haaniella* *erringtoniae* Redtenbacher, 1906 | Heteropteryginae: Heteropterygini | Bacillidae | present study | West Malaysia (culture) |
| *Heteropteryx* *dilatata* (Parkinson, 1798) | Heteropteryginae: Heteropterygini | Bacillidae | [11] | West Malaysia (culture) |
| Korinninae sp. nov. | Korinninae | Aschiphasmatidae [13]  or Prisopodidae [14] | present study | Vietnam |
| *Leiophasma lucubense* (Brancsik, 1893) | Leiophasmatinae | Anisacanthidae | present study | Madagascar |
| *Mearnsiana bullosa* Rehn & Rehn, 1939 | Heteropteryginae: Obrimini | Bacillidae | present study | Philippines (culture) |
| *Melophasma antillarum* (Caudell, 1914) | Pseudophasmatinae: Prisopodini [12] or  Prisopodinae: Paraprisopidini [14] | Pseudophasmatidae [12] or  Prisopodidae [14] | present study | Guadeloupe |
| *Orestes mouhotii* (Bates, 1865) | Heteropteryginae: Datamini | Bacillidae | present study | West Malaysia (culture) |
| *Peruphasma schultei* Conle & Hennemann, 2005 | Pseudophasmatinae: Anisomorphini | Pseudophasmatidae | [11] | Peru (culture) |
| *Phyllium* *siccifolium* (Linnaeus, 1758) | Phylliinae | Phylliidae | [11] | Philippines (culture) |
| *Pseudophasma velutinum* (Redtenbacher, 1906) | Pseudophasmatinae: Pseudophasmatini | Pseudophasmatidae | [11] | Peru (culture) |
| *Pylaemenes guangxiensis* (Bi & Li, 1994) | Heteropteryginae: Obrimini | Bacillidae | present study | China (culture) |
| *Spinonemia chilensis* (Westwood, 1859) | Heteronemiinae: Heteronemiini | Heteronemiidae | [11] | Vicente Perez Rosales, Peulla, Chile |
| *Sungaya* *inexpectata* Zompro, 1996 | Heteropteryginae: Obrimini | Bacillidae | present study | Philippines (culture) |
| Euphasmatodea: ‘Anareolatae’ | | | | |
| *Achrioptera spinosissima* (Kirby, 1891) | Achriopterini | Phasmatidae | present study | Madagascar |
| *Achrioptera fallax* Coquerel, 1861 | Achriopterini | Phasmatidae | present study | Madagascar |
| *Agamemnon cornutus* (Burmeister, 1838) | Cladomorphinae: Hesperophasmatini | Phasmatidae | [11] | West Indies (culture) |
| *Asceles* sp. | Necrosciinae | Diapheromeridae | [17] | Thailand (culture) |
| *Bactrododema* sp. | Palophinae | Diapheromeridae | [11] |  |
| *Carausius* *morosus* (Sinéty, 1901) | Lonchodinae: Lonchodini | Diapheromeridae | [11] | India (culture) |
| *Chondrostethus woodfordi* Kirby, 1896 | Lonchodinae: Lonchodini | Diapheromeridae | [11] | Guadalcanal, Solomon Islands (culture) |
| *Diapheromera* *femorata* (Say, 1828) | Diapheromerinae: Diapheromerini | Diapheromeridae | [11] | Kiowa County, Kansas, USA |
| *Diesbachia* *tamyris* (Westwood, 1859) | Necrosciinae | Diapheromeridae | [17] | Sumatra (culture) |
| *Eurycantha* *calcarata* Lucas, 1869 | Lonchodinae: Eurycanthini | Phasmatidae | [11] | Papua New Guinea (culture) |
| *Eurycnema* *goliath* (Gray, 1834) | Phasmatinae: Phasmatini | Phasmatidae | [11] | Australia (culture) |
| *Extatosoma* *tiaratum* (MacLeay, 1826) | Tropidoderinae: Extatosomatini | Phasmatidae | [11] | Australia (culture) |
| *Hyrtacus* sp. | Lonchodinae: Lonchodini | Diapheromeridae | [11] | Tennant Springs, Northern Territory, Australia |
| *Lopaphus perakensis* (Redtenbacher, 1908) | Necrosciinae | Diapheromeridae | [17] | Vietnam (culture) |
| *Lopaphus sphalerus* (Redtenbacher, 1908) | Necrosciinae | Diapheromeridae | [17] | Vietnam (culture) |
| *Macrophasma* *biroi* (Redtenbacher, 1908) | Phasmatinae: Stephanacridini | Phasmatidae | [11] | Papua New Guinea, Menakfobib |
| *Megacrania batesii* Kirby, 1896 | Platycraninae | Phasmatidae | [11] | Australia |
| *Neohirasea* *hongkongensis* Brock & Seow-Choen, 2000 | Necrosciinae | Diapheromeridae | [17] | China, Hong Kong (culture) |
| *Neohirasea* *maerens* (Brunner, 1907) | Necrosciinae | Diapheromeridae | [17] | Vietnam (culture |
| *Neopromachus doreyanus* (Bates, 1865) | Lonchodinae: Eurycanthini | Diapheromeridae | [11] | Papua New Guinea (culture) |
| *Orxines xiphias* (Westwood, 1859) | Necrosciinae | Diapheromeridae | [11] | Philippines / Java (cultures) |
| *Oxyartes lamellatus* Kirby, 1904^1^ | Necrosciinae | Diapheromeridae | [11] | Vietnam (culture) |
| *Oxyartes* *spinipennis* Carl, 1913 | Necrosciinae | Diapheromeridae | [17] | Vietnam (culture) |
| *Paramenexenus* *laetus* (Kirby, 1904) | Necrosciinae | Diapheromeridae | [17] | Vietnam (culture) |
| *Phaenopharos* *herwaardeni* Hennemann, Conle & Bruckner, 1996 | Necrosciinae | Diapheromeridae | [17] | Thailand (culture) |
| *Phaenopharos* *struthioneus* (Westwood, 1859) | Necrosciinae | Diapheromeridae | [17] | West Malaysia (culture) |
| *Phasmotaenia* sp. | Phasmatinae: Stephanacridini | Phasmatidae | [11] | Malaita, Solomon Islands (culture) |
| *Pseudodiacantha* *macklottii* (De Haan, 1849) | Necrosciinae | Diapheromeridae | [17] | Java (culture) |
| *Pseudosermyle phalangiphora* (Rehn, 1907) | Diapheromerinae: Diapheromerini | Diapheromeridae | [11] | Belize (culture) |
| *Pterinoxylus* *crassus* Kirby, 1899 | Cladomorphinae: Hesperophasmatini | Phasmatidae | [11] | Martinique (culture) |
| *Rhamphosipyloidea* *gorkomi* (Hausleithner, 1990) | Necrosciinae | Diapheromeridae | [17] | Philippines (culture) |
| *Sceptrophasma hispidulum* (Wood-Mason, 1873) | Pachymorphinae: Gratidiini | Diapheromeridae | [11] | Andaman Islands (culture) |
| *Sipyloidea* *sipylus* (Westwood, 1859) | Necrosciinae | Diapheromeridae | [11] | Madagascar (culture) |
| *Thaumatobactron guentheri* Hennemann & Conle, 1997 | Lonchodinae: Eurycanthini | Phasmatidae | [11] | Papua New Guinea |
| *Trachythorax* *maculicollis* (Westwood, 1848) | Necrosciinae | Diapheromeridae | [17] | Burma (culture) |
| *Zehntneria mystica* Brunner, 1907 | Pachymorphinae: Gratidiini | Diapheromeridae | [11] | South Africa |

*Novel sequence data generated in the present study are deposited on GenBank under the accession numbers KP300885–KP300930. Sequence data from previous studies can be accessed under accession numbers FJ474100–FJ474403 (for [11]), KJ024376–KJ024575 (for [17]), AY121129–AY121186 and AY125216–AY125326 (for [18]).
